# New Incoherent Autofluorescence/Fluorescence System for Early Detection of Lung Cancer

**DOI:** 10.1155/DTE.5.71

**Published:** 1999

**Authors:** Martin Leonhard

**Affiliations:** Karl Storz Gmbh & Co. Marketing New Technologies Mittelstr. 8 Tuttlingen D-78532 Germany

## Abstract

A new autofluorescence (AF) system for bronchoscopy that operates as compact as a conventional white light bronchoscopy system is described. The system is also capable of white light illumination and excitation of aminolevulinic acid (ALA) induced fluorescence. Changing between white light and (auto-) fluorescence mode is easy and always possible. Broad band excitation with blue light (AF: 380–460 nm; ALA 380–440 nm) delivers high intensity illumination at the distal end of the bronchoscope (AF typically 50 mW). A special optical observation technique makes the AF directly visible to the eye instead of indirect techniques used in other AF systems. A compact (160 g)and sensitive (typically 0.2 lux) camera can be used for documentation.


*Diagnostic and Therapeutic Endoscopy*, Vol. 5, pp. 71–75
Reprints available directly from the publisher
Photocopying permitted by license only

© 1999 OPA (Overseas Publishers Association) N.V.
Published by license under
the Harwood Academic Publishers imprint,
part of The Gordon and Breach Publishing Group.
Printed in Singapore.

### New Incoherent Autofluorescence/Fluorescence System for Early Detection of Lung Cancer

MARTIN LEONHARD*

*KARL STORZ GmbH & Co., Marketing New Technologies, Mittelstr. 8, D-78532 Tuttlingen, Germany*

**A new autofluorescence (AF) system for bronchoscopy that operates as compact as a conventional white light bronchoscopy system is described. The system is also capable of white light illumination and excitation of aminolevulinic acid (ALA) induced fluorescence. Changing between white light and (auto-) fluorescence mode is easy and always possible. Broad band excitation with blue light (AF: 380–460 nm; ALA 380–440 nm) delivers high intensity illumination at the distal end of the bronchoscope (AF typically 50 mW). A special optical observation technique makes the AF directly visible to the eye instead of indirect techniques used in other AF systems. A compact (160 g) and sensitive (typically 0.2 lux) camera can be used for documentation.**

*Keywords:* ALA, Autofluorescence, Bronchoscopy, D-Light AF, Early detection, Lung cancer

### INTRODUCTION

Perception depends upon the detection technique. What we see is the result of the brilliant possibilities of the human eye and the human brain. Vision enables us to orient and survive in our natural surroundings. However, only a fraction of all optical information is processed in the natural visual chain.

Going beyond normal vision is not necessary for living but can reveal a lot more information about the object being observed. Cleverly capturing this additional information opens the door to new analytical and diagnostic techniques. One of these new techniques is to reveal information hidden inside tissue to discriminate between suspect tumor tissue and normal tissue.

### Basics of Fluorescence Imaging

Common visual pictures correlate the remission spectrum of an object with their physical properties: e.g. color, shape, texture, orientation and motion. The natural objective is to recognize known structures. Incident light $L(\lambda, x)$ as a function of wavelength $\lambda$ and space $x$, which usually is, "white" sunlight, illuminates the scenery and the objects modulate the incident light due to different absorption and scattering characteristics $A(\lambda, x)$. The

* Tel.: +49 7461 708 521. Fax: +49 7461 708 292. E-mail: karlstorz-marketing@karlstorz.de.

remitted scattered light $I_{A,S}(\lambda, x)$ has the same wavelength $\lambda_r$ as the incident light $\lambda_i$.

$$I_{A,S}(\lambda, x) = A(\lambda, x) \cdot L(\lambda, x) \quad \lambda_r = \lambda_i = \lambda.$$

For example, blood absorbs light in the blue and green part and remits the remaining spectrum; the color of blood is described as red. This type of modulation is the dominant process which gives us orientation in our natural surrounding and in day-to-day life.

Fluorescence in contrast is an additional effect that contributes to remission and changes the wavelength of incident light. Fluorescence $F(\lambda_{Fl}; \lambda_i; x)$ correlates the wavelength of the incident light $\lambda_i$ and the fluorescence light $\lambda_{Fl}$ and therefore provides additional information. Generally speaking the fluorescent light $I_{Fl}$ is red shifted.

$$I_{Fl}(\lambda_{Fl}, x) = \int F(\lambda_{Fl}; \lambda_i; x) \cdot L(\lambda_i; x)\, d\lambda_i \quad \lambda_{Fl} > \lambda_i.$$

Thus the overall intensity $I(\lambda, x)$ becomes

$$I(\lambda, x) = I_{A,S}(\lambda, x) + I_{Fl}(\lambda_{Fl}, x)$$

and is generally dominated by absorption and scattering effects, because fluorescence is weak.

By employing a simple trick we can make fluorescence visible, i.e. we have to eliminate the dominating scattering contribution to realize the fluorescence. This is possible due to the spectral separation of incident and fluorescent light. By blocking the wavelength of the incident light in the observation pathway we eliminate disturbing scattering light and reveal the fluorescence.

Fluorescence originates in chromophores which can be intrinsic (autofluorescence, AF) or added to the tumor tissue (xenofluorescence).

### Biophysical Effects of Autofluorescence

To evoke AF in the visible range, special molecules or chromophores are required. Various proteins are known to display fluorescence when excited in the near UV or with blue light. These different chromophores are embedded in complex matrices and provide different micro environments to the chromophores. Excitation and fluorescence spectra of the dyes involved are typical broad band spectra. This effect can be used in the clinical application of tissue intrinsic (auto-) fluorescence.

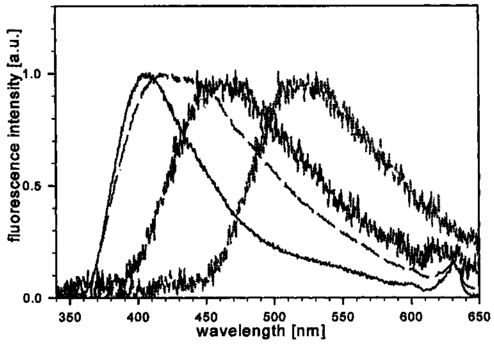

FIGURE 1 Normalized fluorescence spectra of various tissue chromophores in aqueous solution excited at 308 nm (from left to right: Collagen I, Collagen II, NADH, $FADH_2$).

Figure 1 displays several molecules which are expected to play an active role in AF when excited with a XeCl-Laser at 308 nm [1]. This representation is chosen for a more complete display of the spectra. Excitation with the KARL STORZ D (Diagnosis)-Light AF cuts off the short-wavelength part of the spectra.

The penetration depth of light is wavelength dependant and increases with wavelength in the visible range. Blue light penetration depth is limited to a fraction of a millimeter but is significantly deeper than UV light penetration depth. Therefore only the very top layers of tissue can be involved in optical effects. Fluorescence is suspected to originate in the submucosal layer.

The modulation responsible for contrasting early stage tumors is not revealed in detail. One effect is modified metabolic activity in tumor cells which effects also fluorescence characteristics. Another effect is tissue optical based and comes from the epithelium which absorbs light depending on the thickness of the layer (Fig. 2) according to Lambert–Beer's law. Malignant alterations display thickened epithelium and therefore appear darker under AF detection. Additionally, only a smaller

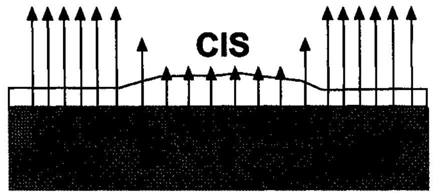

FIGURE 2 Tissue optical effect revealing early tumor stages, e.g. carcinoma *in situ* (CIS). Excitation at 380–460 nm; detection at 470–800 nm (schematical drawing).

amount of submucosal tissue can be illuminated due to limited penetration depth.

### Biophysical Effects of ALA-induced Fluorescence

Inhalation of the endogeneous substance 5-aminolevulinic acid (ALA) leads to a tumor sensitive fluorescence marking. The heme metabolic pathway is made use of and transforms ALA into protoporhyrin IX (PPIX) which is accumulated in tumor cells. PPIX shows an absorption maximum at 410 nm and fluorescence with broad peaks at 635 and 705 nm. Details are available in this volume (Huber *et al.*) and elsewhere [2].

The method of ALA-induced detection of early tumor stages is successfully applied with D-Light excitation for detection of superficial bladder cancer [3], malignant glioma [4] and in various other fields.

### MATERIALS AND METHODS

*History* The incoherent light system used to induce fluorescence is the new KARL STORZ D-Light AF system (KARL STORZ, Tuttlingen, Germany) which is based on a technology developed for early detection of superficial bladder cancer [3]. This incoherent diagnostic approach for ALA-induced fluorescence was found to be clinically more convenient than a krypton-ion laser which originally was used for illumination [5]. Rapid technological innovation has created a powerful bronchoscopy system capable of (auto-) fluorescence detection. No contrast agent is required with the autofluorescence technology.

### D-Light AF System

The D-Light AF system is handy, weighs less than 10 kg and is capable of three different illumination modes: (1). Conventional white light mode; (2) AF mode; (3) ALA-induced fluorescence mode which is described elsewhere [2]. The system is based on a 300-W Xenon lamp with special optics to focus high intensities of light into a liquid light guide, which is optimized for blue light transmission. The modes can easily be switched any time by a footswitch. In the AF mode output wavelength is between 380 and 460 nm; in the ALA mode between 380 and 440 nm. Blue light output power for AF at the distal end of a KARL STORZ broncho-fiberscope is typically 50 mW.

### Observation

Both rigid telescopes and flexible fiberscopes are compatible with the KARL STORZ D-Light AF system. Critical to this method of tumor detection is the use of the correct observation technique as mentioned above which is realized in these special endoscopes. The specially designed endoscopes contain a filter wheel with three different positions for white light mode (a), for AF mode (b) and optionally for ALA-induced fluorescence mode (c). Position (a) does not contain any filter to allow standard white light bronchoscopy, whereas positions (b) and (c) contain observation filters that block the incident light properly for AF or ALA mode, respectively. For optimal contrast, specificity, illumination and plasticity a small amount of the blue excitation light bypasses the detection filters. With this trick, observation of biopsy forceps or other instruments is ensured even in (auto-) fluorescence mode.

The complete procedure can be performed entirely with D-Light AF and the special bronchoscope, and allows detection with the naked eye which is known for its high dynamic range. The technique used is direct imaging instead of indirect as used in other approaches [6].

The rigid telescopes used are Hopkins bronchoscopic telescopes with 5.5 mm diameter and different directions of view (0° and 30°). They are optimized for fluorescence observation as described above. The 0° telescope provides extremely high luminous intensity so that white light conditions can almost be matched in the AF mode to allow perfect imaging and observation.

The fiberscopes were remodeled, improved and adapted for (auto-) fluorescence, with both a 5.0 mm (11001 BI) and 6.4 mm version (11004 BI) available. The distal tip has a upward range of 180° movement and a downward range of 100°/80°, respectively. Instrument channels are 2.3/2.8 mm, respectively. Optical quality is brilliant in both (auto-) fluorescence and white light mode.

### Documentation

To allow proper documentation special endocameras are available (Telecam SL PDD, Tricam SL PDD, both KARL STORZ, Tuttlingen, Germany). These CCD-cameras have a optimized optical system and allow additional on-chip integration for the fluorescence modes. With a typical integration time of 1/15 s the Telecam SL PDD achieves sensitivity better than 0.2 lux. Luminous sensitivity can be increased by one order of magnitude. The endocamera weighs about 160 g and therefore has no need for any holding device. The camera can nicely communicate with the D-Light AF system to synchronize illumination and detection modes. For blue light illumination (both AF and ALA mode) the camera is switched to the blue light mode from the white light mode which is used as for conventional endocameras. The blue light mode allows the extended on-chip-integration as described above and has a specially adapted built-in color balancing function for fluorescence mode to match with the colors that are visually perceived. The complete system is compact and provides flexibility by allowing examination of a fluorescence finding with the white light as often as necessary. Additional image storage devices can be easily configured and added.

Clinical experience is reported in this issue and elsewhere [2,7–9]. The system is compact (Fig. 3), easy to handle, economic and gives brilliant images for all illumination modes. The KARL STORZ AF system is CE-marked and commercially available for the European market and selected countries.

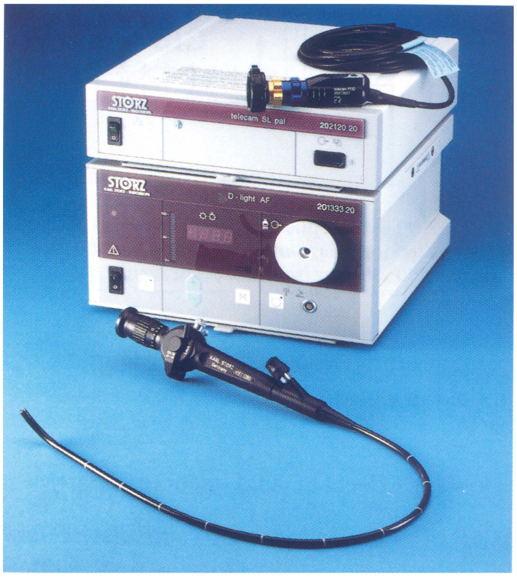

FIGURE 3 Karl Storz fluorescence detection system D-Light AF with fiberbronchoscope 11004 BI and Telecam SL PDD.

### *Acknowledgements*

We kindly thank our clinical and scientific partners Professor Dr. Häußinger, Dr. Stanzel (Fachklinik Munich-Gauting) and PD Dr. Huber (Klinikum Innenstadt, University of Munich) and the University of Munich at Großhadern Laser Research Department with Dr. Baumgartner, Dr. Stepp and Dipl. Biol. Pichler for brilliant work.

### *References*

[1] Pichler, J. and Stepp, H. Spectral data reproduced with kind permisson of Laser Research Department, University Munich at Großhadern, 1998.

[2] Baumgartner, R., Huber, R.M., Schulz, H. *et al.* Inhalation of 5-aminolevulinic acid: a new technique for fluorescence detection of early stage lung cancer. *J. Photochem. Photobiol. B* 1996; **36**: 169–174.

[3] Kriegmair, M., Stepp, H., Steinbach, P. *et al.* Fluorescence cystoscopy following intravesical instillation of 5-aminolevulinic acid: A new procedure with high sensitivity for detection of hardly visible urothelial neoplasis. *Urol. Int.* 1995; **55**: 190–196.

[4] Stummer, W., Stocker, S., Wagner, S., Stepp, H. *et al.* Intraoperative detection of malignant gliomas by 5-aminolevulinic acid-induced porphyrin fluorescence. *Neurosurgery* 1998; **42**: 518–526.

[5] Kriegmair, M., Baumgartner, R., Knüchel, R. *et al.* Fluorescence photodetection of neoplastic urothelial lesions following intravesical instillation of 5-aminolevulinic acid. *Urology* 1994; **44**: 836–841.

[6] Lam, S., Kennedy, T., Unger, M. *et al.* Localization of bronchial intraepithelial neoplastic lesions by fluorescence bronchoscopy. *Chest* 1998; **113**: 696–702.

[7] Stanzel, F., Häußinger, K., Pichler, J. and Sauer, W. The result of a pilot study with an autofluorescence bronchoscopy system. *10th World Congress for Bronchology*, Budapest 1998.

[8] Stanzel, F., Häußinger, K., Sauer, W. and Pichler, J., ALA-induced fluorescence and autofluorescence bronchoscopy in detection of lung cancer. *Europ. Resp. J.* 1998; **12**, suppl. 28: 132s.

[9] Huber, R.M., Gamarra, F., Leberig, A. *et al.* Stellenwert der Fluoreszenzmethoden in der bronchologischen Diagnostik: Früherkennung des Bronchialkarzinoms möglich? *Atemw Lungenkrkh* 1995; **21**: 558–561.

